# The Impact of Ginger Mouthwash on Pregnant Women with Stress and Gingivitis by Measuring Different Salivary Biomarker Levels

**DOI:** 10.5152/eurasianjmed.2026.251093

**Published:** 2026-02-25

**Authors:** Batool Abbas Tareq, Athraa Ali Mahmood, Hashim Mueen Hussein

**Affiliations:** 1 Department of Oral Surgery and Periodontics, Mustansiriyah University College of Dentistry, Baghdad, Iraq; 2 Department of Conservative Dentistry, Mustansiriyah University College of Dentistry, Baghdad, Iraq

**Keywords:** Chlorhexidine, cortisol, diaphragmatic breathing, ginger mouthwash, gingivitis, lactoferrin, pregnancy, stress

## Abstract

**Background:**

Gingivitis is common among pregnant women due to hormonal changes, and stress can increase the severity. Chlorhexidine (CHX) is the standard treatment for gingivitis, but its side effects limit its use during pregnancy. Ginger has an anti-inflammatory and antioxidant effect. The research aims to evaluate ginger mouthwash’s impact on gingival health, cortisol, and lactoferrin (LF) levels in pregnant women with stress and gingivitis compared to distilled water and CHX.

**Methods:**

The completed research was a parallel 3-arm triple-blind randomized clinical investigation. The study included 45 pregnant women with stress and gingivitis. Clinical periodontal indicators (bleeding on probing [BOP], plaque index [PI], and gingival index [GI]) were examined at baseline visit and after 7 days of using mouthwash, diaphragmatic breathing, and progressive muscle relaxation (PMR). Salivary cortisol and LF levels were measured, and a comparison was made before and after treatment. The participants answered a “visual analog scale-based questionnaire” at the second visit.

**Results:**

Significantly reduced in BOP, PI, and GI in all interventions, but ginger and CHX had a greater significant effect compared to placebo. All mouthwashes, diaphragmatic breathing, and PMR significantly reduced cortisol and LF concentrations. However, the responses to the questionnaire showed that ginger and CHX had significant differences in Q1 and Q3, while nonsignificant differences in Q2, Q4, Q5, and Q6.

**Conclusion:**

Ginger mouthwash achieved CHX-comparable reductions in BOP, GI, and PI and decreased cortisol and LF concentrations after 1 week from baseline without adverse effects, highlighting a safe, natural alternative during pregnancy.

**Main Points**

- Ginger mouthwash demonstrated a significant improvement in gingival inflammation among pregnant women with stress and a significant decrease in salivary cortisol and lactoferrin.
- Ginger mouthwash was as effective as chlorhexidine (CHX) in reducing gingival inflammation and may serve as a natural alternative to CHX for treatment of gingivitis.
- No adverse effects were reported with ginger mouthwash use, indicating it is safe during pregnancy.

## Introduction

Gingivitis is a type of periodontal disease that affects soft tissue and can be reversed by appropriate treatment.^1^ The primary causative factor during the establishment of gingivitis is “dental plaque,” and the severity of inflammation can be influenced by hormonal and other factors.^2^ Plaque-induced gingivitis in pregnancy is a commonly observed periodontal disease that occurs in the second or third months of pregnancy and has a prevalence rate from 30% to 100%.^3^ Furthermore, periodontal diseases are a risk factor for adverse pregnancy outcomes, including preterm birth, fetal growth restriction, preeclampsia, low birth weight, and gestational diabetes.^4^

In addition, research has provided evidence on the impact of stress on the formation of periodontal conditions.^5^ Risk factors such as deficient sleep, deficient dietary intake, oral hygiene deficiency, and smoking are indirectly linked with stress and periodontal disease by contributing to the buildup of plaque, leading to compromised periodontal health.^6^

Depending on the severity of gingivitis, it can be treated through regular tooth brushing, scaling, root planing, and mouthwash that contains chlorhexidine (CHX).^7^ Chlorhexidine mouthwash has side effects (e.g., taste alteration, staining) that might be particularly problematic for pregnant women who are already experiencing nausea or heightened sensory sensitivity. Herbal medicine is useful for the prevention and treatment of periodontal disease.^8^ One example is ginger, which has antimicrobial properties that can suppress the secretion of inflammatory mediators like prostaglandins and leukotrienes by focusing on the enzymes cyclooxygenase and 5-lipoxygenase, respectively. In addition, ginger works against gram-negative anaerobic bacteria and is significant for gingivitis and periodontitis.^9^ Based on evidence, ginger could be a benign and effective treatment for women suffering from nausea and vomiting of pregnancy.^10^

In addition, non-pharmacological stress-reduction techniques have been beneficial for improving both psychological and physiological outcomes during pregnancy, such as diaphragmatic breathing and progressive muscle relaxation (PMR).

This research not only explores a natural, cost-effective therapeutic approach but also addresses the multidimensional challenges faced by this population.

## Material and Methods

### Design of the Study

The completed study was a parallel-3-arm triple-blind randomized clinical investigation that was conducted between January and April 2025 at Gynecology and Obstetrics Hospital. This study was approved by the Ethics Committee of the College of Dentistry of the University (approval number: MUOSU-202125, date: January 1, 2025). This study was registered at clinicaltrials.gov, “XX NIHR” in 2025 under identifier number “US NIHR” in 2025 under identifier number (NCT06792812), https://www.clinicaltrial.gov/study/NCT06792812. 

Patients received a consent form to voluntarily agree to an overview of the study’s goals and purposes.

### Sample Size Calculation

The sample size was calculated by G-Power 3.1.9.7 with a statistical power of 80%, α-error probability set at 0.05 (two-sided), assuming a medium effect size (*f* = 0.25), 3 groups, and 2 measurements; under all these conditions, the sample size is about 45 participants (15 allocated to each group). To avoid dropout, 3 samples were taken in each group.

### Inclusion Criteria

Pregnant women with stress and gingivitis with intact periodontium, in the second trimester, aged 20-30 years, good general health without systemic disease, a minimum of 20 natural teeth present, and no periodontal therapy has been received in the last 3 months.

### Exclusion Criteria

Pregnant with periodontitis who currently use any mouthwash, who reject the form of informed consent, with dental implants and orthodontic or prosthodontic appliances, or any retentive factor of dental plaque.

### Blinding, Random Allocation, and Interventions

Each sample had the same probability of being allocated to any intervention protocol sequence. The examiner block-randomized patient enrollment into 3 groups: A, B, or C, representing each group’s intervention. Using random numbers in Microsoft Excel, the groups of participants were rearranged on a 1 : 1 : 1 basis.

All participants, examiner, and analysts did not know the type of mouthwash by placing it in bottles that were not transparent and were assigned sequential number codes (A, B, and C) by a doctor not included in this study.

Group A takes distilled water.

Group B takes ginger mouthwash Pasta del Capitano, Italy.

Group C takes CHX mouthwash 0.12% KIN Gingival, Barcelona, Spain.

### Clinical Procedure

**Figure 1.**
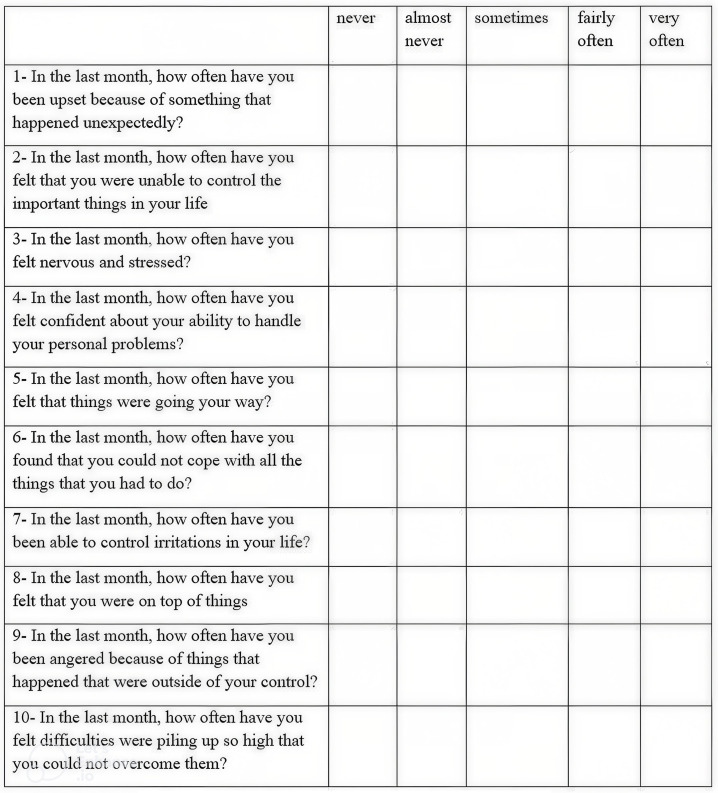
The PSS questionnaire used in the study.

At first visit (baseline), all examined subjects were requested to complete a questionnaire that included the subject’s name, age, medical and dental history, and the previous history of periodontal treatment. Then, the symptoms of stress were assessed by a translated Arabic version of the perceived stress scale (Figure 1).

Then, saliva was collected by unstimulated (drooling method) before periodontal indicators recording bleeding on probing (BOP), plaque index (PI), gingival index (GI), probing pocket depth (PPD) by the principal examiner. Before saliva collection, subjects were instructed not to eat any type of food for at least 2 hours, and a 15-second rinse with drinking water was performed to remove residual food debris, microbial contaminants, and desquamated epithelial cells.^11^ Bleeding on probing was examined by the insertion of a periodontal probe and monitoring the presence of bleeding. O’Leary PI was measured, where the probe was used to confirm the presence of plaque. Gingival index measures gingival inflammation depending on color, consistency, and BOP. All pregnant women then received coded bottles of mouthwash, a toothbrush with medium-hard bristles, and a toothpaste, both supplied by Colgate-Palmolive (New York, USA). The pregnant women were advised to perform tooth brushing twice a day. After 30 minutes from tooth brushing, the pregnant women were rinsed with 10 mL of mouthwash, without dilution, for 1 minute, and a post-rinse restriction of 30 minutes, without food or drink, was recommended. A dentist does not perform professional dental hygiene procedures or periodontal intervention. All pregnant women were instructed to decrease stress by a combination of diaphragmatic breathing and PMR.

The second visit was 7 days from the baseline visit; a salivary sample was collected again. Then, periodontal indicators (BOP, PI, and GI) were taken. Finally, all pregnant women answered a questionnaire of VAS designed to evaluate their experience with the product.

### Analysis of Salivary Biomarker

The concentrations of biomarkers in salivary samples were assessed using Enzyme-linked immunosorbent assay kits, which were supplied by Cloud-Clone Corp, Houston, USA.

### Statistical Analysis

Descriptive Analysis: Mean rank for qualitative variables, while mean and SD for quantitative variables, Graphs: Cluster chart bars.

Inferential analysis: Intraclass correlation coefficient test correlation between 2 related points, Shapiro–Wilk test for evaluating whether the quantitative variable followed a normal distribution, 1-way analysis of variance statistical difference of quantitative variables among k independent groups using Tukey Honestly Significant Difference (equal variance) and Dunnett’s T3 (unequal variance), and Paired *t*-test the difference between 2 related points.

## Results

### Study Population

**Figure 2.**
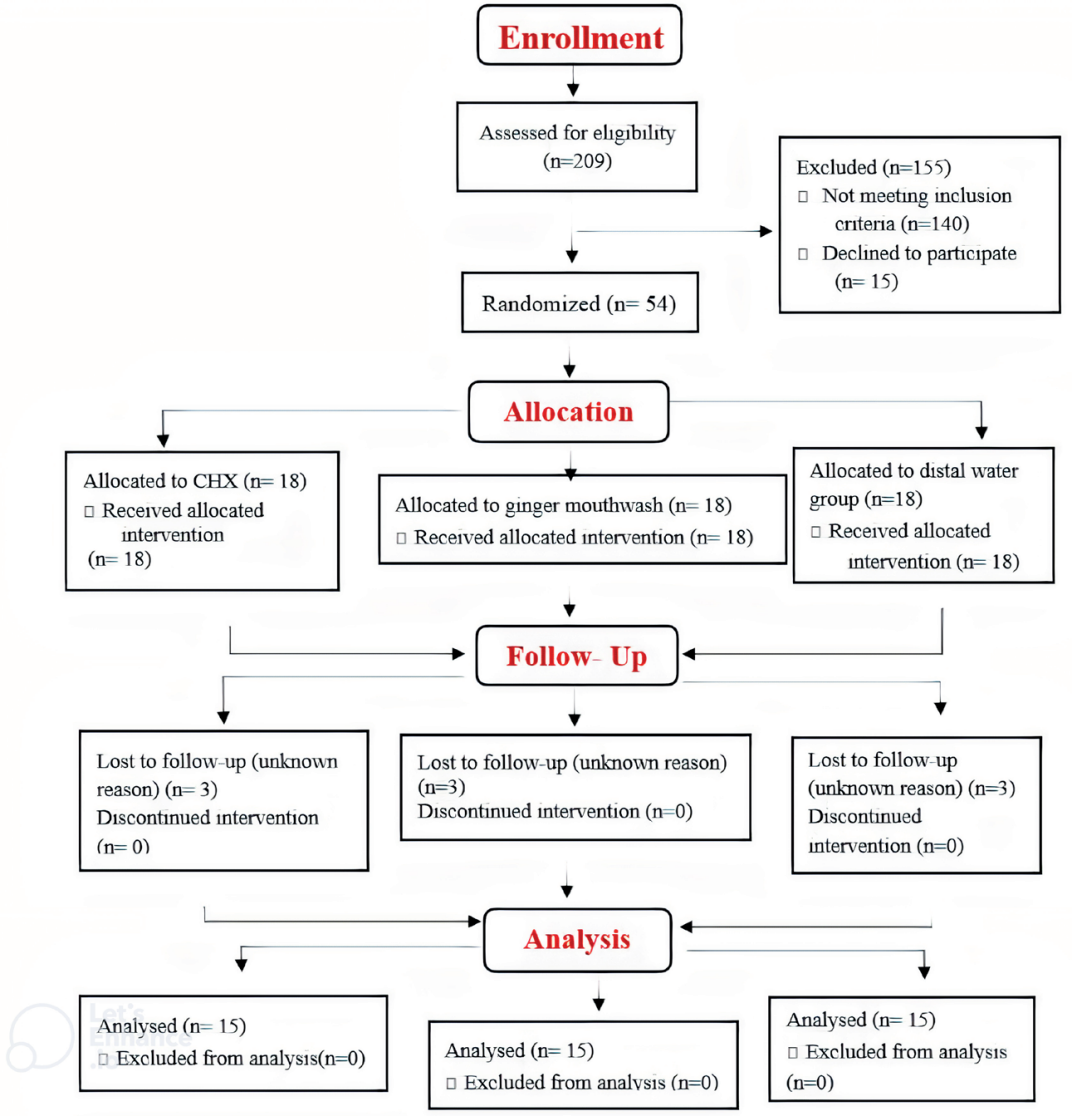
Flow chart diagram of the study.

About 209 pregnant women were initially examined in the completed study based on the inclusion and exclusion criteria. Only 54 pregnant women were enrolled in the study and randomly assigned in equal numbers to 3 groups (n = 18). Three pregnant women from each group exited the study as a result of participants not adhering to their appointment dates. Fifteen pregnant women in each group finished the study (Figure 2).

### Analysis of Clinical Indicators of Periodontal Status and Salivary Cortisol and Lactoferrin

**Figure 3.**
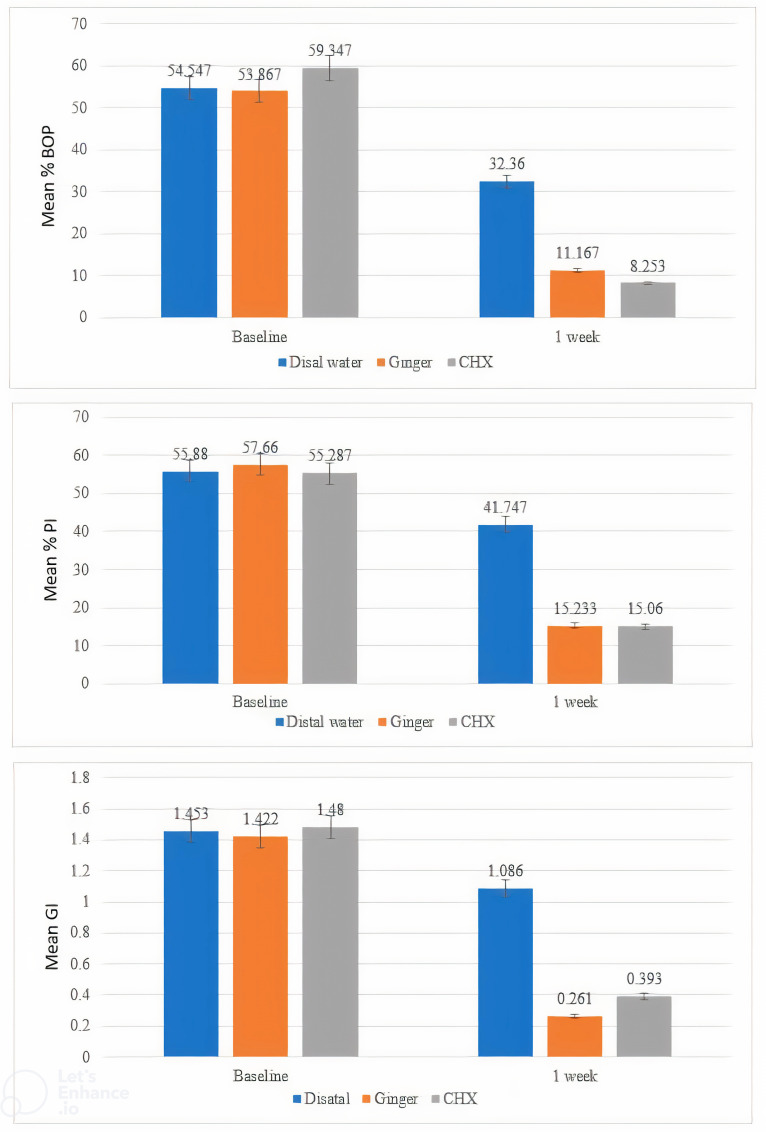
Bar chart for the distribution of mean values of BOP%, PI%, and GI among groups and intervals.

**Figure 4.**
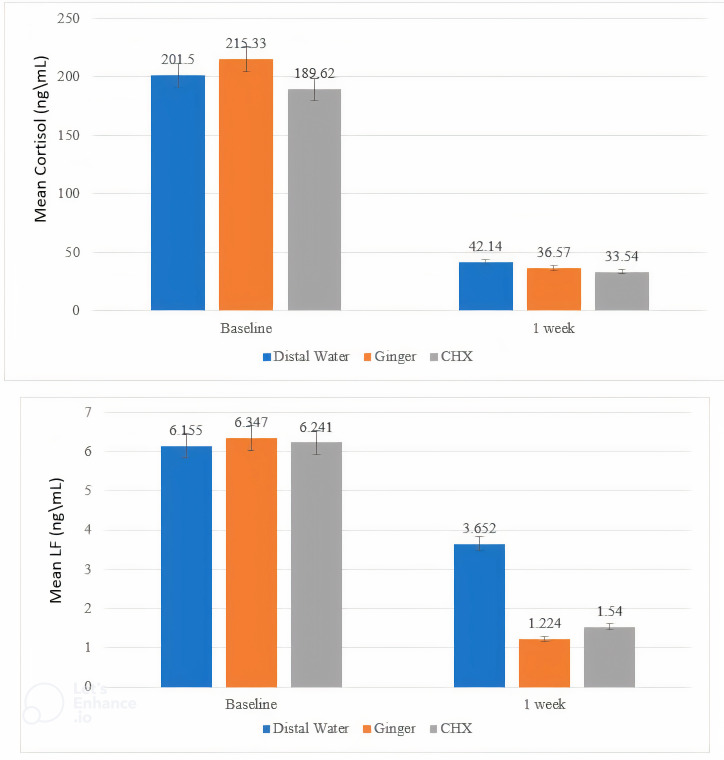
Bar chart for the distribution of mean values of Cortisol and LF among groups and intervals.

The main outcome of the study is the improvement in BOP. Reduction of PI and GI represents another important outcome. At the initial visit, there were no statistically significant differences in BOP, PI, GI, cortisol, and lactoferrin (LF) in all groups. The results of the second visit noted a statistically significant difference in all groups in BOP, PI, GI, cortisol, and LF (Table 1). The ginger group had the highest effect size, followed by CHX and placebo groups (Table 2). Furthermore, the descriptive statistics for BOP%, PI%, and GI mean values are demonstrated graphically in Figure 3. And the descriptive statistics for cortisol and LF mean values are presented graphically in Figure 4.

In Table 3, multiple pairwise comparisons were made between groups for the mean %BOP, %PI, GI, cortisol, and LF values at the second visit. A statistically significant difference was noted between the distilled water and the ginger groups. Furthermore, there was a significant difference between the distilled water and CHX groups in BOP, PI, GI, and LF. While a non-significant difference was noted between the CHX and ginger groups in BOP, PI, GI, cortisol, and LF. Furthermore, there was a significant difference between the distilled water and ginger groups in cortisol.

### Visual Analog Scale Questionnaire

In Table 4, significant differences were noted in the mean of responses to all questions. In (Table 5) (Q1), the participants rated ginger mouthwash to have a significantly more flavorful taste than CHX. (Q2), participants rated CHX mouthwash to have a significantly longer duration of taste compared to ginger. (Q3), participants rated ginger mouthwash to have a significantly greater effect on the test than CHX. Regarding the utilization of mouth rinse comforted (Q4), rinsing time (Q5), and (Q6), the impact on plaque reduction, it showed that ginger was not significantly different from CHX.

## Discussion

Mouth rinsing represents one of the most commonly used and convenient methods to deliver anti-plaque agents.^12^ Chlorhexidine mouthwash remains the most chemotherapeutic agent due to its superior efficacy in controlling oral biofilm formation, but long-term usage of CHX has different negative effects.^13^ In the completed study, CHX was used for only 1 week to minimize adverse effects in pregnant women.

This study introduces a novel approach, being the first clinical trial to explore the efficacy of ginger mouthwash in managing both gingivitis and stress in pregnant women compared to a CHX mouthwash.

The group taking ginger mouthwash shows a significant decline in BOP, PI, and GI due to ginger’s anti-inflammatory and antioxidant properties. A matching result was seen in a randomized clinical investigation by Anshula et al,^9^ which showed a significant decline in the GI and PI scores in children after using ginger mouthwash. Another study showed that Zingiber officinale essential oil mouthwash is anti-inflammatory in decreasing BOP and PI.^14^

The group taking distilled water noted a significant reduction in BOP, PI, and GI due to patient motivation and education about oral hygiene and its effect on health, and the correct brushing technique to remove dental plaque.^15^

Cortisol concentrations were previously found to increase in subjects with gingivitis.^16^ Consistent with this research, high concentrations of cortisol in the 3 groups at the initial visit and after 1 week of using mouthwash significantly decreased in all groups, in contrast to the values recorded at baseline. The decrease in cortisol observed in this study is important, as cortisol is a biomarker of psychological stress. The present study is the first study of the impact of ginger on cortisol in pregnant women.

All participants performed diaphragmatic breathing and PMR under the same conditions, frequency, and duration. These contributed to a decrease in cortisol levels across all groups. This agrees with a study by Tragea et al,^17^ which shows significant benefits from the diaphragmatic breathing techniques in the psychological state of pregnant women. Another study by Weschenfelder et al^18^ shows that the PMR is widely used as a simple, economical approach to reduce stress during pregnancy. Promoting relaxation and physiological balance may enhance resistance to inflammation and support oral hygiene, assisting in lowering gingival inflammation. However, because these exercises were applied equally in all groups, the differences observed among the groups were primarily attributed to mouthwash interventions.

Ginger mouthwash may indicate a stress reduction due to the active ingredients in ginger, such as 6-gingerol and shogaol, which modulate the hypothalamic-pituitary-adrenal axis.^19^ Furthermore, CHX improved periodontal health may contribute to reduced stress. This is attributed to CHX’s ability to suppress bacterial biofilm and inflammation,^20^ which can lower secretion of glucocorticoids like cortisol.

Lactoferrin concentrations were previously found to increase in subjects with gingivitis.^21^ Consistent with this research, high concentrations of LF in the 3 groups at the initial visit and after 1 week of using mouthwash significantly decreased the level in all groups. This research represents the first assessment of the influence of ginger and CHX-mouthrinses on LF.

A decreased level of LF after using ginger mouthwash may be due to its anti-inflammatory and antimicrobial activity. Gingerol and shogaol, key bioactive components, reduce the secretion of TNF-α.^19^ The decreasing level of LF after using CHX mouthwash may be due to the effect of CHX on decreasing gingival inflammation by lowering the level of TNF-α, indirectly reducing neutrophil activation.^22^

At the final stage of the study, participants’ feedback regarding the mouthwash was collected. Q1 participants disliked CHX taste, as in most studies.^23^ Participants liked the ginger mouthwash; this result agrees with the previous research.^24^ (Q2), the taste of CHX remained longer than distilled water and ginger. This agreed with the results of previous research.^25^ (Q3), ginger mouthwash had a significantly greater effect on the taste than CHX and placebo. Ginger decreases nausea during pregnancy.^10^ Benefits from decreased nausea included enhanced taste and nutrition. (Q4), participants who used ginger mouthwash felt comfortable without any hypersensitivity or burning sensation, and this agrees with previous studies.^9^ (Q5), the response to ginger and CHX was significantly nondifferent. Finally (Q6), this results from ginger mouthwash causing a decrease in the number of lactobacillus count.^9^

This study has a limitation related to its 1-week follow-up period. A short duration does not allow evaluation of long-term effects, recurrence, or safety. Longer follow-up studies (3-4 weeks) are required to confirm whether the effects are sustained. Other limitations include that only systemically healthy pregnant women were included, those who had a bad mood, had difficulty communicating due to hormonal changes, were reminded by phone to use mouthwash and a stress reduction program every day, and were lost to follow-up in the second visit.

## Conclusion

The study’s main finding is the effect of ginger mouthwash on treating gingivitis in pregnant women with stress. It improves periodontal parameters (BOP, PI, and GI) and decreases salivary biomarkers (cortisol and LF) after 1 week from the baseline period. While ginger mouthwash appears as effective as CHX for short-term management, long-term studies with microbiological profiling and hormonal monitoring are still required.

## Figures and Tables

**Table 1.** Descriptive Data and Statistical Tests Among Time and Groups

		**Distilled Water**	**Ginger**	**CHX**	***P***
**BOP**	BOP% baseline visit (mean ± SD)	54.547 ± 6.296	53.867 ± 13.441	59.347 ± 8.085	.257
	BOP% second visit (mean ± SD)	32.360 ± 9.033	11.167 ± 13.025	8.2533 ± 1.148	**<.001 **
	Paired *t*-test	11.913	9.584	25.249	
	*P*	**<.001 **	**<.001 **	**<.001 **	
	Effect size	3.076	5.885	5.194	
**PI**	PI baseline visit (mean ± SD)	55.880 ± 4.605	57.660 ± 1.588	55.287 ± 3.368	.154
	PI second visit (mean ± SD)	41.747 ± 8.379	15.233 ± 2.821	15.060 ± 2.575	**<.001 **
	Paired *t*-test	6.864	49.465	30.124	
	*P*	**<.001 **	**<.001 **	**<.001 **	
**GI**	GI baseline visit (mean ± SD)	1.453 ± 0.113	1.422 ± 0.077	1.480 ± 0.108	.297
	GI second visit (mean ± SD)	1.086 ± 0.168	0.261 ± 0.170	0.393 ± 0.252	**<.001 **
	Paired *t*-test	13.189	22.221	19.936	
	*P*	**<.001 **	**<.001 **	**<.001 **	
**Cortisol**	Baseline visit Mean ± SD	201.50 ± 13.92	215.33 ± 38.17	189.62 ± 55.50	.219
	Second visit Mean ± SD	42.14 ± 10.96	36.57 ± 2.26	33.54 ± 3.17	**.004 **
	Paired *t*-test	9.863	18.317	11.115	
	*P*	**<.001 **	**<.001 **	**<.001 **	
**LF**	Baseline visit Mean ± SD	6.155 ± 0.260	6.347 ± 0.183	6.241 ± 0.239	.084
	Second visit Mean ± SD	3.652 ± 1.363	1.224 ± 0.292	1.540 ± 0.332	**<.001 **
	Paired *t*-test	7.683	77.178	38.961	
	*P*	**<.001 **	**<.001 **	**<.001 **	

*P* < .05 statistically significant, *P* ≥ .05 not significant.

BOP, bleeding on probing; CHX, chlorohexidine; GI, gingival index; LF, lactoferrin; PI, plaque index.

**Table 2.** Effect Size Among Groups

	**Effect Size**		
	**Distilled Water**	**Ginger**	**CHX**
BOP	3.076	5.885	5.194
PI	1.772	12.772	7.778
GI	3.405	5.738	5.147
Cortisol	1.88	4.73	2.87
LF	1.98	19.93	10.06

BOP, bleeding on probing; CHX, chlorohexidine; GI, gingival index; LF, lactoferrin; PI, plaque index.

**Table 3.** Multiple Pair-wise Comparisons of BOP%, GI, Cortisol, and LF Using Tukey HSD and PI% Using Dunnett’s T3 After 1 Week Among Groups

	**(I) Groups**	**(J) Groups**	**Mean Difference (I-J)**	***P***	**95% CI**	
					**Lower Bound**	**Upper Bound**
BOP	Distilled water	Ginger	21.193	**<.001 **	13.053	29.333
		CHX	24.107	**<.001 **	15.967	32.247
	Ginger	CHX	2.913	.662	-5.227	11.053
PI	Distilled water	Ginger	26.513	**<.001 **	20.507	32.520
		CHX	26.513	**<.001 **	20.713	32.660
	Ginger	CHX	0.173	.173	-2.325	2.672
GI	Distilled water	Ginger	0.825	**<.001 **	0.647	1.003
		CHX	0.693	**<.001 **	0.515	0.871
	Ginger	CHX	-0.132	.182	-0.310	0.046
Cortisol	Distilled Water	Ginger	5.573	.071	-0.385	11.531
		CHX	8.601	**.003 **	2.644	14.559
	Ginger	CHX	3.028	.440	-2.929	8.986
LF	Distilled water	Ginger	2.429	**<.001 **	1.695	3.162
		CHX	2.112	**<.001 **	1.379	2.846
	Ginger	CHX	-0.316	.552	-1.050	0.418

*P* < 0.05 statistically significant. *P* ≥ 0.05 not significant.

BOP, bleeding on probing; CHX, chlorohexidine; GI, gingival index; LF, lactoferrin; PI, plaque index.

**Table 4.** Descriptive and Statistical Tests of VAS Among Groups

	**Distilled Water **			**Ginger**			**CHX**			**Kruskal–Wallis**	***P***
	**Mean**	**±SD**	**MR**	**Mean**	**±SD**	**MR**	**Mean**	**±SD**	**MR**		
Q1	9.000	0.655	33.10	8.200	1.082	25.10	6.400	1.502	10.80	23.983	**<.001 **
Q2	5.133	0.640	14.43	6.133	1.302	24.93	6.400	0.986	29.63	11.662	**.003 **
Q3	6.400	0.828	16.43	7.600	0.632	32.63	6.600	0.986	19.93	14.347	**.001 **
Q4	9.400	0.632	35.90	7.667	1.113	18.50	7.200	1.265	14.60	23.538	**<.001 **
Q5	7.933	1.100	31.63	6.800	0.862	16.77	7.133	0.640	20.60	11.547	**.003 **
Q6	6.533	1.407	14.33	8.133	1.506	27.77	8.067	0.961	26.90	10.253	**<.001 **

*P* < .05 statistically significant, *P* ≥ .05 not significant.

CHX, chlorohexidine; MR, mean rank; Q, question.

**Table 5.** Multiple Pairwise Comparisons of VAS Between Groups Using the Wilcoxon Signed-rank Test Adjusted by the Dunn-Bonferroni Method

**Variables**	**CHX-Ginger**	**CHX-Distilled Water**	**Ginger-Distilled Water**
Q1	**0.006 **	**<0.001 **	0.249
Q2	0.907	**0.003 **	0.064
Q3	**0.014 **	0.999	**0.001 **
Q4	0.999	**<0.001 **	**0.001 **
Q5	0.999	**0.045 **	**0.003 **
Q6	0.999	**0.022 **	**0.013 **

*P* < .05 statistically significant, *P* ≥ .05 not significant.

CHX, chlorohexidine; Q, question.

## Data Availability

The data that support the findings of this study are available on request from the corresponding author.
